# Mixed-Frequency rTMS Rapidly Modulates Multiscale EEG Biomarkers of Excitation–Inhibition Balance in Autism Spectrum Disorder: A Single-Case Report

**DOI:** 10.3390/brainsci15121269

**Published:** 2025-11-26

**Authors:** Alptekin Aydin, Ali Yildirim, Olga Kara, Zachary Mwenda

**Affiliations:** 1 Clinical Psychology, Atlantic International University, Honolulu, HI 96813, USA; alptekinaydin@hotmail.com; 2 Deepsynaps, Oxford OX4 4GP, UK; 3 Cosmos Healthcare, London EN2 6DR, UK; olgakara@hotmail.com (O.K.); zack123@gmail.com (Z.M.)

**Keywords:** autism spectrum disorder, rTMS, mixed-frequency stimulation, EEG biomarkers, excitation–inhibition balance, aperiodic slope, long-range temporal correlations, functional connectivity

## Abstract

**Background**: Repetitive transcranial magnetic stimulation (rTMS) is an established neuromodulatory method, yet its multiscale neurophysiological effects in autism spectrum disorder (ASD) remain insufficiently characterized. Recent EEG analytic advances—such as spectral parameterization, long-range temporal correlation (LRTC) assessment, and connectivity modeling—enable quantitative evaluation of excitation–inhibition (E/I) balance and network organization. **Objective**: This study aimed to examine whether an eight-session, EEG-guided mixed-frequency rTMS protocol—combining inhibitory 1 Hz and excitatory 10 Hz trains individualized to quantitative EEG (qEEG) abnormalities—produces measurable changes in spectral dynamics, temporal correlations, and functional connectivity in a pediatric ASD case. **Methods**: An 11-year-old right-handed female with ASD (DSM-5-TR, ADOS-2) underwent resting-state EEG one week before and four months after intervention. Preprocessing used a validated automated pipeline, followed by spectral parameterization (FOOOF), detrended fluctuation analysis (DFA), and connectivity analyses (phase-lag index and Granger causality) in MATLAB (2023b). No inferential statistics were applied due to the single-case design. The study was conducted at Cosmos Healthcare (London, UK) with in-kind institutional support and approved by the Atlantic International University IRB (AIU-IRB-22-101). **Results**: Post-rTMS EEG showed (i) increased delta and reduced theta/alpha/beta power over central regions; (ii) steeper aperiodic slope and higher offset, maximal at Cz, suggesting increased inhibitory tone; (iii) reduced Hurst exponents (1–10 Hz) at Fz, Cz, and Pz, indicating decreased long-range temporal correlations; (iv) reorganization of hubs away from midline with marked Cz decoupling; and (v) strengthened parietal-to-central directional connectivity (Pz→Cz) with reduced Cz→Pz influence. **Conclusions**: Mixed-frequency, EEG-guided rTMS produced convergent changes across spectral, aperiodic, temporal, and connectivity measures consistent with modulation of cortical E/I balance and network organization. Findings are preliminary and hypothesis-generating. The study was supported by in-kind resources from Cosmos Healthcare, whose authors participated as investigators but had no influence on analysis or interpretation. Controlled trials are warranted to validate these exploratory results.

## 1. Introduction

Autism spectrum disorder (ASD) is increasingly conceptualized as a disorder of neural circuit homeostasis, in which the delicate balance between excitatory glutamatergic and inhibitory γ-aminobutyric acid (GABA)ergic transmission is disrupted [1]. Converging genetic, post-mortem, and in vivo evidence implicates alterations in synaptic proteins—such as neuroligins, neurexins, SHANK, and mTOR-regulated pathways—that shift local networks toward hyper- or hypo-excitability, contributing to both behavioral manifestations and epilepsy comorbidity [2,3,4].

Electrophysiology provides a non-invasive window into this excitation–inhibition (E/I) balance. Beyond canonical oscillations, the aperiodic “background” of the EEG power spectrum follows a 1/f-like decay in which the slope and offset index the aggregate synaptic drive of neuronal populations. Flatter slopes have been linked to cortical hyper-excitability, whereas steeper slopes reflect stronger inhibitory tone [5,6,7]. These measures are reproducible across species and developmental stages and sensitively track pharmacological or developmental changes in E/I regulation [8,9]. In ASD, several recent reports describe flatter aperiodic exponents or elevated broadband offsets, consistent with increased excitatory drive [6,10,11].

Complementary temporal-domain metrics, such as the Hurst exponent derived from detrended fluctuation analysis (DFA), quantify long-range temporal correlations (LRTC) that depend on recurrent excitatory–inhibitory interactions [12]. Abnormal LRTC patterns in ASD suggest circuit-level dysregulation and reduced temporal flexibility [13]. Graph-theoretical analyses of functional connectivity further reveal a motif of short-range over-connectivity with reduced long-range coordination [14,15,16] reinforcing that E/I imbalance alters both local gain control and network topology. Together, these multiscale EEG measures—spectral, aperiodic, temporal-complexity, and connectivity—provide an integrated biomarker framework for probing circuit dysfunction in ASD [17].

Repetitive transcranial magnetic stimulation (rTMS) delivers trains of magnetic pulses that transiently entrain cortical neurons and induce plasticity-like after-effects lasting minutes to months [18,19,20]. The physiological outcome is frequency-dependent: high-frequency (>5 Hz) stimulation generally increases excitability, whereas low-frequency (≤1 Hz) tends to suppress it, likely through mechanisms akin to long-term depression and GABAergic potentiation [21,22]. rTMS has received regulatory approval for several neuropsychiatric conditions and is under active investigation in neurodevelopmental disorders associated with altered E/I balance, including ASD and ADHD [23,24,25]. Emerging studies report variable yet measurable behavioral and electrophysiological changes, and safety profiles remain favorable in pediatric populations when International Federation of Clinical Neurophysiology (IFCN) standards are followed [26,27,28]. Despite growing clinical interest, mechanistic understanding of rTMS effects in ASD is limited. Few studies integrate stimulation with advanced neurophysiological readouts, and most focus narrowly on power spectra or event-related potentials, omitting aperiodic, temporal-complexity, or network measures [7,29,30]. This gap constrains the development of data-driven, individualized neuromodulation protocols. Because rTMS effects propagate across distributed cortical systems, examining multiscale EEG biomarkers offers a valuable avenue for understanding its network-level impact [31,32].

Electrophysiology provides a non-invasive window into this excitation–inhibition (E/I) balance. Beyond canonical oscillations, the aperiodic “background” of the EEG power spectrum follows a 1/f-like decay in which the slope and offset index the aggregate synaptic drive of neuronal populations. Flatter slopes have been linked to cortical hyper-excitability, whereas steeper slopes reflect stronger inhibitory tone [5,6] These measures are reproducible across species and developmental stages and sensitively track pharmacological or developmental changes in E/I regulation [8,9]. In ASD, several recent reports describe flatter aperiodic exponents or elevated broadband offsets, consistent with increased excitatory drive [10,11].

Complementary temporal-domain metrics, such as the Hurst exponent derived from detrended fluctuation analysis (DFA), quantify long-range temporal correlations (LRTC) that depend on recurrent excitatory–inhibitory interactions [12]. Abnormal LRTC patterns in ASD suggest circuit-level dysregulation and reduced temporal flexibility [13]. Graph-theoretical analyses of functional connectivity further reveal a motif of short-range over-connectivity with reduced long-range coordination [14,15] reinforcing that E/I imbalance alters both local gain control and network topology. Together, these multiscale EEG measures spectral, aperiodic, temporal-complexity, and connectivity provide an integrated biomarker framework for probing circuit dysfunction in ASD.

Repetitive transcranial magnetic stimulation (rTMS) delivers trains of magnetic pulses that transiently entrain cortical neurons and induce plasticity-like after-effects lasting minutes to months [18]. The physiological outcome is frequency-dependent: high-frequency (>5 Hz) stimulation generally increases excitability, whereas low-frequency (≤1 Hz) tends to suppress it, likely through mechanisms akin to long-term depression and GABAergic potentiation [21,22]. rTMS has received regulatory approval for several neuropsychiatric conditions and is under active investigation in neurodevelopmental disorders associated with altered E/I balance, including ASD and ADHD [23,24]. Emerging studies report variable yet measurable behavioral and electrophysiological changes, and safety profiles remain favorable in pediatric populations when International Federation of Clinical Neurophysiology (IFCN) standards are followed [26,27]. Despite growing clinical interest, mechanistic understanding of rTMS effects in ASD is limited. Few studies integrate stimulation with advanced neurophysiological readouts, and most focus narrowly on power spectra or event-related potentials, omitting aperiodic, temporal-complexity, or network measures [29,30]. This gap constrains the development of data-driven, individualized neuromodulation protocols. Because rTMS effects propagate across distributed cortical systems, examining multiscale EEG biomarkers offers a valuable avenue for understanding its network-level impact [31].

### Significance and Study Context

This exploratory case study was conducted at Cosmos Healthcare (London, UK) with in-kind institutional support and approved by the Atlantic International University IRB (AIU-IRB-22-101). We implemented a mixed-frequency, EEG-guided rTMS protocol in a pediatric ASD case, selecting stimulation targets based on baseline quantitative EEG (qEEG) abnormalities rather than standardized landmarks an approach consistent with principles of personalized, hypothesis-generating neuromodulation [33]. What distinguishes this work from prior single-case reports is the integration of multiple EEG biomarkers aperiodic spectral features, periodic oscillatory activity, DFA-derived LRTC, and both symmetric and directed connectivity within a unified analytic pipeline. The goal was not to test clinical efficacy but to demonstrate the feasibility and neurophysiological interpretability of EEG-guided rTMS in ASD and to generate mechanistic hypotheses for future controlled studies.

## 2. Methods

The participant (pseudonym: P2) was an 11-year-old right-handed female referred to the Neuromodulation Clinic at Cosmos Healthcare (London, UK) by a community pediatric neurologist for assessment of persistent social-communication difficulties, circumscribed interests (notably military aircraft), and episodic irritability that had not improved with behavioral therapy. Early developmental history indicated delayed expressive language two-word phrases emerged around age three while gross and fine motor milestones were age-appropriate. Early language delay is a recognized predictor of social-communication challenges in ASD [34]. Family history revealed broader neurodevelopmental traits: a maternal uncle with Asperger-like characteristics and an older sibling with dyslexia. There was no first-degree family history of epilepsy or major psychiatric disorder, consistent with broader-phenotype clustering [35].

The patient was born full-term at 39 + 5 weeks by elective caesarean section with no perinatal complications (Apgar scores = 9 and 10). At intake, P2 attended a mainstream school with one-to-one support. Strengths included advanced receptive vocabulary and exceptional visual memory for schematic systems (e.g., subway maps) [36]. Reported challenges involved social reciprocity, pragmatic language use, and pronounced sensory hypersensitivity to fluorescent lighting an observation consistent with atypical sensory gating in ASD [37]. The patient had no history of psychotropic medication use. Melatonin (3 mg) was taken intermittently for sleep onset delay [38]. Past medical history was notable only for mild eczema, managed with emollients. Ophthalmologic evaluation identified accommodative lag; corrective lenses (+1.25 D) were prescribed. Neurological examination was normal (cranial nerves, reflexes, coordination, gait). Conners-3 parent/teacher questionnaires indicated clinically significant inattention consistent with the DSM-5-TR specifier “with accompanying ADHD-like traits,” though full ADHD criteria were not met. Diagnosis of ASD was confirmed using the Autism Diagnostic Observation Schedule, Second Edition (ADOS-2, Module 3), with a total score of 15 (above diagnostic threshold) and a Calibrated Severity Score (CSS) of 7, corresponding to moderate severity [39].

This work was conducted as a prospective, hypothesis-generating case study with in-kind institutional support from Cosmos Healthcare (access to EEG and rTMS equipment, clinical space, and limited staff time). The research was independently approved by the Institutional Review Board of Atlantic International University (Approval Code: AIU-IRB-22-101). The protocol was not registered as a clinical trial and did not claim therapeutic intent. Parents provided written informed consent after being explicitly informed that rTMS for ASD is an experimental, non-standard intervention used solely for exploratory research. Potential risks—including transient scalp discomfort, fatigue, mood changes, and a low seizure risk (<0.1% per session)—were discussed in accordance with IFCN safety guidelines. All procedures adhered to the Declaration of Helsinki and CARE case report guidelines [40]. The EEG preprocessing pipeline referenced the MADE and FASTER toolboxes, which are validated for developmental EEG data [17,28,41].

### 2.1. Intervention

All rTMS sessions were conducted at Cosmos Healthcare (London, UK) in a shielded, quiet environment adapted for neuromodulation research. The clinic provided in-kind access to equipment and space, but had no role in study design, data analysis, or interpretation.

Prior to the first session, a standard TMS safety screening was completed, and 30 dB earplugs were used throughout all procedures. Stimulation parameters and safety monitoring adhered strictly to International Federation of Clinical Neurophysiology (IFCN) pediatric safety guidelines [23,26]. A trained clinician continuously observed the participant during each session, and no physiological monitoring abnormalities were recorded.

#### 2.1.1. Stimulation Targeting and Protocol Design

Cortical targets were determined using the international 10–20 EEG coordinate system, guided by each region’s baseline quantitative EEG (qEEG) profile rather than by MRI-based neuronavigation. This individualized, EEG-informed approach allowed region-specific modulation of hyper- or hypo-active cortical areas. The mixed-frequency protocol combined both inhibitory and excitatory trains:

Inhibitory trains (1 Hz, 90% RMT): delivered to midline cortical hubs (Fz, Cz, Pz) that displayed elevated high-frequency power and increased aperiodic offset, to reduce cortical hyper-excitability.

Excitatory trains (10 Hz, 100% RMT): delivered to frontopolar and lateral temporal sites (FP1, FP2, F3, T6) that showed reduced beta activity or hypoconnectivity on baseline qEEG, to enhance cortical engagement.

Each session followed a fixed stimulation order with 60 s inter-train intervals to prevent coil overheating and minimize cumulative fatigue. Across the eight sessions, a total of 43,200 pulses were delivered. The coil position was stabilized with a custom 3D-printed frame to ensure reproducibility across sessions. Stimulation was applied using a MagPro X100 stimulator (MagVenture A/S, Farum, Denmark) with a Cool-B65 figure-of-eight coil.

#### 2.1.2. Scientific Rationale

The combination of inhibitory and excitatory trains was intended to rebalance regional network asymmetries downregulating hyperactive midline hubs while upregulating hypoactive lateral and frontopolar regions. Although homeostatic or metaplastic interactions between stimulation blocks cannot be ruled out, the consistent stimulation sequence and spatial separation of targets reduce potential confounding.

#### 2.1.3. Adverse Events and Tolerability

Adverse events were systematically monitored and recorded using CTCAE v5.0 grading criteria (Table 1). All reported events were mild (Grade 1), transient, and resolved spontaneously without medical intervention or lasting sequelae. No serious or unanticipated adverse events occurred.

### 2.2. Stimulation, EEG Acquisition & Analysis

Stimulation was delivered using a MagPro X100 system (MagVenture A/S, Denmark) with a Cool-B65 figure-of-eight coil with active cooling. The resting motor threshold (RMT) was determined at each session via standard motor-evoked potential criteria. Intensities were set at 90% RMT for 1 Hz trains and 100% RMT for 10 Hz trains. A fixed stimulation order was maintained across all sessions. Coil positioning was stabilized with a custom 3D-printed support frame to ensure reproducibility. Although continuous physiological monitoring (ECG, oximetry) was not employed, a trained clinician remained present throughout, and the patient was visually observed for safety. The protocol adhered to International Federation of Clinical Neurophysiology (IFCN) guidelines for pediatric rTMS safety [23,26]. 

Across eight mixed-frequency rTMS sessions (43,200 pulses), only mild Grade 1 adverse events—scalp discomfort, transient headache, fatigue, and brief irritability—were reported, all resolving spontaneously without sequelae (Table 2). No serious adverse events occurred. Transient Grade 1 events (Common Terminology Criteria for Adverse Events, CTCAE v5.0) were observed and resolved spontaneously or with minimal intervention (Table 2). Resting-state EEG was recorded for 10 min (eyes open) at two time points: baseline (one week before rTMS) and follow-up (four months post-intervention). Recordings were performed in a shielded, sound-attenuated room under identical conditions.

Electrodes were placed using a 10–10 montage with a Brain Products actiCAP (Brain Products GmbH, Munich, Germany). The online reference was FCz; ground was AFz. Signals were amplified and digitized at 500 Hz using a Mitsar-EEG 202 amplifier. Impedances were kept <10 kΩ. Preprocessing was conducted using a validated automated pipeline [41] in MATLAB, with custom routines in R 4.3.2 and Python 3.11.6.

Steps included:

1. Filtering: 1 Hz high-pass, 40 Hz low-pass (4th order Butterworth).
2. Channel rejection: flat channels removed; noisy channels flagged by the FASTER algorithm (Nolan et al., 2010, [28]), using thresholds for variance, correlation, and Hurst exponent.
3. Artifact correction: Independent Component Analysis (ICA, Infomax) removed ocular/muscle artifacts.
4. Epoching: Cleaned data segmented into 2 s epochs; epochs exceeding ±125 µV were rejected.
5. Re-referencing: average reference applied post-cleaning.

For DFA, continuous 400 s artifact-free segments were used to preserve temporal dynamics.

PSD was estimated per epoch using Welch’s method (0.5 s windows, 50% overlap). This window length prioritizes variance reduction at higher frequencies but reduces delta-band resolution. To address this trade-off, delta results were interpreted in conjunction with aperiodic slope/offset measures. PSDs were normalized to total 1–40 Hz power and log-transformed.

Normalized PSD values were averaged within canonical bands: delta (1–4 Hz), theta (4–8 Hz), alpha (8–13 Hz), beta (13–30 Hz), and low gamma (30–40 Hz). Topographies were plotted to visualize spatial distributions.

The FOOOF algorithm [5], implemented in FieldTrip, decomposed spectra into aperiodic and periodic components over 1–40 Hz. Both with and without the knee parameter were tested; results converged across settings. Reported results are from knee-free fits, with confirmatory analyses noted in Methods. Aperiodic-adjusted spectra were used to examine true oscillatory peaks. DFA was applied to 400 s cleaned broadband signals (1–40 Hz) and to band-specific envelopes. The signal was cumulatively summed, detrended at 20 logarithmically spaced scales, and RMS fluctuations calculated. The Hurst exponent was estimated as the slope of log-log RMS vs. window size. Directed connectivity was estimated using time-domain Granger causality. Bivariate AR models (order = 15; ~750 ms embedding) were fit to 2 s epochs with the armorf function (BSMART toolbox). Model stability was checked (all eigenvalues within unit circle), and residual whiteness was confirmed (Ljung–Box test). Pilot tests with orders 10–20 yielded similar results, supporting robustness. GC values were expressed as the log-ratio of residual variances.

## 3. Results

### 3.1. Spectral Power Across the Scalp

To examine how mixed-frequency rTMS affected cortical dynamics, we first compared power spectra across all scalp electrodes at baseline (pre-rTMS) and follow-up (post-rTMS). This analysis characterized frequency-specific changes in oscillatory activity independent of behavioral interpretation.

Following rTMS, a broad increase in delta-band power and reductions in theta, alpha, and beta power were observed across posterior and right-lateral electrodes. The most pronounced attenuation appeared over the central scalp (Cz), particularly within the alpha and beta ranges. These localized spectral reductions are spatially consistent with stimulation at the midline sites included in the protocol. As this is a single-case, exploratory analysis, findings are interpreted descriptively and not as evidence of efficacy.

### 3.2. Result 1—Aperiodic and Periodic Components of Cortical Excitation–Inhibition Balance

To better interpret these spectral changes, we decomposed each power spectrum into its aperiodic (1/f) and periodic (oscillatory) components. In EEG, total power at a given frequency reflects both broadband aperiodic activity and narrowband rhythmic activity. Because broadband (1/f) power can obscure or inflate oscillatory peaks, spectral parameterization enables a more physiologically specific assessment of oscillatory modulation [5,8,42].

The aperiodic component is characterized by two parameters: the exponent (slope), reflecting how rapidly power decreases with frequency, and the offset (intercept), representing broadband power at a reference frequency.

Lower exponents (flatter slopes) correspond to more desynchronized, excitatory-dominant cortical states, whereas steeper slopes are associated with increased inhibitory tone and low-frequency dominance (Voytek & Knight, 2015, [6]). Given that low-frequency rTMS (<1 Hz) typically reduces cortical excitability [21,22], we hypothesized that the 1 Hz midline stimulation component could alter the aperiodic slope and offset at Fz, Cz, and Pz—the principal midline targets in this protocol. Decomposition of these channels therefore provided an index of putative excitation–inhibition modulation and isolated true oscillatory changes from broadband spectral shifts. Separating the total spectrum into periodic and aperiodic elements also allowed inspection of canonical oscillatory power (delta, theta, alpha, beta) uncontaminated by broadband changes. This approach facilitated clearer interpretation of frequency-specific alterations, ensuring that observed differences reflected genuine rhythmic modulation rather than shifts in background spectral slope or offset.

**Figure 1:**
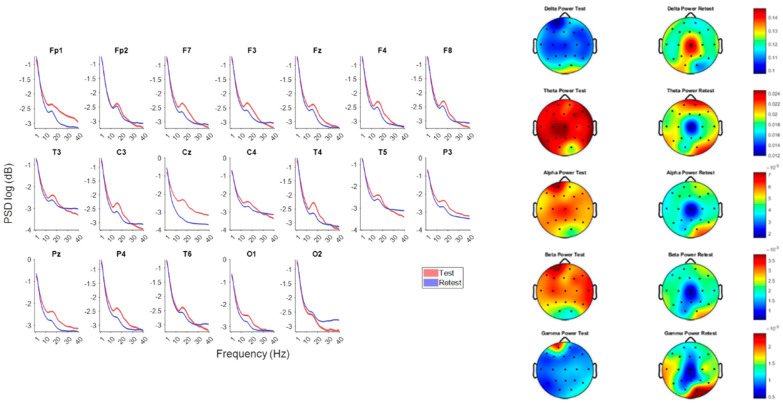
Spectral power changes across electrodes and frequency bands before and after rTMS. (**Left**): Normalized power spectral density (PSD) estimates at each electrode for pre-rTMS (blue) and post-rTMS (red) sessions. PSDs were normalized to total 1–40 Hz power and log-transformed to emphasize differences at higher frequencies. (**Right**): Topographical maps showing PSD distributions within canonical frequency bands (delta 1–4 Hz, theta 4–8 Hz, alpha 8–13 Hz, beta 13–30 Hz, gamma 30–40 Hz).

At all three midline electrodes, the post-rTMS spectra exhibited higher aperiodic offsets and steeper exponents (slopes) relative to baseline. The most pronounced change occurred at Cz, where the offset increased from 1.35 to 2.58 (+91%) and the exponent from 1.82 to 2.52 (+38%). This concurrent rise in offset and slope, together with the increase in low-frequency (delta) power seen in Figure 1, indicates a shift in overall spectral shape toward greater low-frequency dominance. The offset elevation does not imply a broadband power gain across all frequencies but likely results from the stronger delta activity near the 1 Hz reference point used in the log–log aperiodic fit.

**Figure 2:**
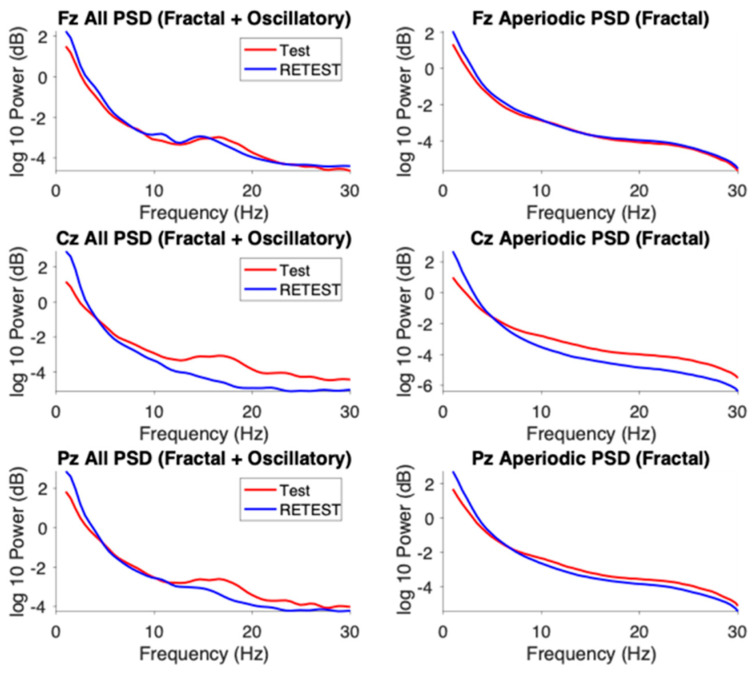
Decomposition of power spectra into aperiodic (fractal) and oscillatory components at midline electrodes before and after 1 Hz TMS. (**Left**): PSD estimates (log10-transformed) at frontal (Fz), central (Cz), and parietal (Pz) electrodes during test (red) and retest (blue) sessions, showing the combined aperiodic and oscillatory components of the EEG signal. (**Right**): Corresponding aperiodic (fractal-only) components of the PSD after separation using spectral parameterization.

To better interpret these spectral changes, we decomposed each power spectrum into its aperiodic (1/f) and periodic (oscillatory) components (Figure 2). This decomposition enabled clearer visualization of how mixed frequency rTMS altered the aperiodic slope and offset across midline electrodes.

To better isolate oscillatory components, the power spectra were adjusted for the aperiodic contribution (FOOOF-based subtraction) and compared with the unadjusted spectra across canonical bands (Table 3).

- Delta (1–4 Hz): Power increased after rTMS at all sites, most strongly at Cz; the enhancement was larger after aperiodic adjustment.
- Theta (4–8 Hz): A modest increase appeared at Fz, whereas Cz and Pz showed a small decrease after adjustment, revealing spatial heterogeneity.
- Alpha (8–13 Hz): Adjusted spectra uncovered a clear post-rTMS alpha enhancement at all midline sites—particularly Cz—despite an apparent reduction in the unadjusted data, suggesting that broadband shifts had masked true rhythmic increases.
- Beta (13–30 Hz): Power decreased at Cz and Pz in both analyses, more prominently once the aperiodic component was removed.
- Theta/Beta Ratio: The ratio rose across Fz, Cz, and Pz, with the largest increase at Fz and Cz after aperiodic correction, indicating differential modulation of low- and high-frequency activity.

Overall, adjusting for the 1/f background revealed several directionally distinct changes compared with unadjusted estimates. For instance, alpha power appeared stable before correction but increased markedly afterward, whereas theta changes became more evident in the adjusted spectra. These contrasts highlight the interpretive importance of separating periodic oscillations from broadband aperiodic activity, particularly in protocols expected to influence both excitatory–inhibitory balance and spectral slope [5].

### 3.3. Result 2—Long-Range Temporal Correlations (LRTC)

The aperiodic component of the EEG spectrum reflects scale-free, or fractal, neural activity characterized by a 1/f distribution of power with no dominant frequency. To examine these dynamics in the time domain, we applied Detrended Fluctuation Analysis (DFA) to estimate Hurst exponents, which quantify long-range temporal correlations (LRTC). The Hurst exponent (H) describes the slope of the linear relationship between fluctuation magnitude and temporal scale on a log–log plot:

- **H = 0.5** → uncorrelated (white-noise-like) activity,
- **H > 0.5** → persistent correlations (large changes followed by large changes),
- **H < 0.5** → antipersistent or rapidly alternating activity.

For this analysis, 400 s artifact-free, continuous EEG segments were extracted from Fz, Cz, and Pz at baseline and follow-up. DFA was computed for broadband (1–40 Hz) and band-specific signals.

**Figure 3:**
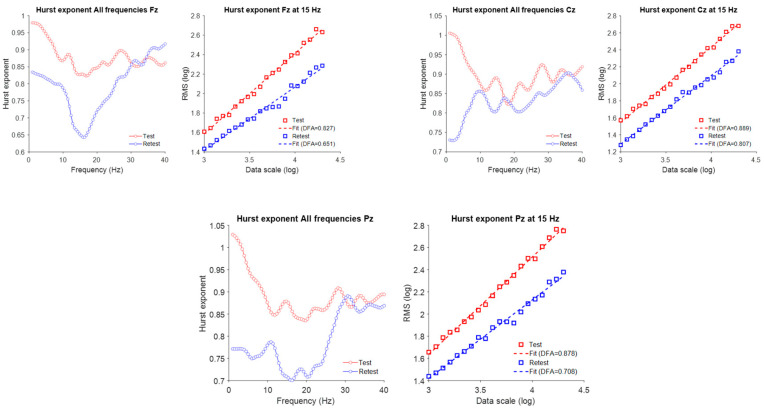
Changes in long-range temporal correlations (LRTC) following 1 Hz TMS across midline EEG channels. (**Left**): Frequency-resolved Hurst exponent curves for Fz, Cz, and Pz derived from EEG amplitude envelopes across 1–40 Hz, plotted for test (red) and retest (blue) sessions. (**Right**): (DFA) plots at 15 Hz (within the beta frequency range), illustrating the log–log relationship between window size and RMS fluctuation. Linear fits (dashed lines) indicate the slopes used to derive the Hurst exponent (DFA α).

In all three channels, the Hurst exponent decreased consistently in the low-frequency range (1–10 Hz) following rTMS. In the mid-frequency range (10–30 Hz), the exponent decreased at frontal (Fz) and parietal (Pz) electrodes but remained stable at the central (Cz) electrode. As Figure 3 mentioned, these region-specific modulations of LRTC within the mid-frequency range may reflect differential responsiveness of associative cortical areas to subthreshold neuromodulation. In contrast, the high-frequency (>30 Hz) Hurst exponent did not substantially change at any of the analyzed channels, indicating that gamma-band temporal dynamics remained largely unaffected by rTMS, at least over the measured longer time scales.

### 3.4. Result 3: Impact of Neuromodulation on Network Connectivity Patterns

Building upon the spectral and fractal analyses, we next assessed how rTMS influenced functional network organization by examining phase-based connectivity within canonical frequency bands. This allowed us to investigate alterations in large-scale connectivity patterns and changes in network hub dynamics resulting from neuromodulation.

**Figure 4:**
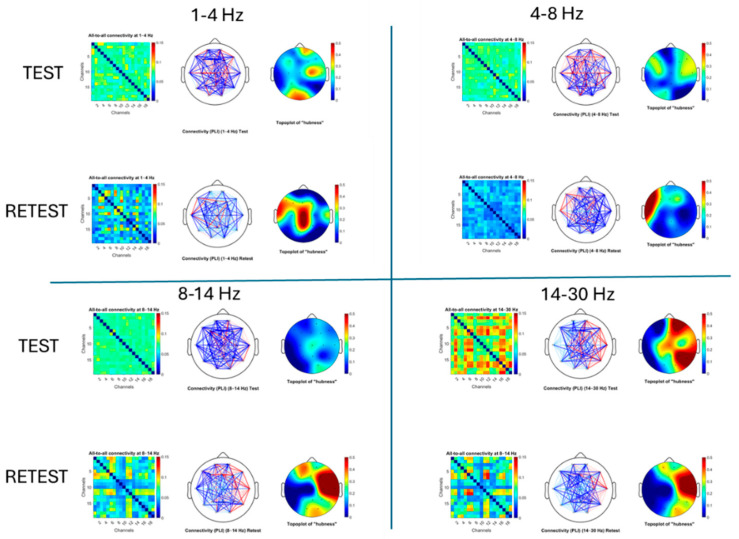
Connectivity patterns across four canonical frequency bands (1–4 Hz, 4–8 Hz, 8–14 Hz, and 14–30 Hz) before (Test) and after (Retest) rTMS. (**Left panels**): All-to-all connectivity matrices computed using phase lag index (PLI). (**Middle panels**): Circular graph representations illustrating the network connectivity structure. (**Right panels**): Scalp topographies showing nodal “hubness,” quantified via graph theoretical metrics, highlighting regions with higher network centrality. Table 4 shows channel numbers and names.

Figure 4 reveals frequency- and region-specific changes in functional connectivity and network topology following rTMS. In the delta (1–4 Hz) band, connectivity and hubness increased at retest, particularly over central and posterior regions, suggesting enhanced synchronization at slow frequencies. In the theta (4–8 Hz) band, overall connectivity decreased at retest, with these reductions observed predominantly over posterior and central rather than frontal areas.

**Table 4:** Channel numbers and names.

Channel No.	Channel Name
1	FP1
2	FP2
3	F7
4	F3
5	FZ
6	F4
7	F8
8	T3
9	C3
10	CZ
11	C4
12	T4
13	T5
14	P3
15	PZ
16	P4
17	T6
18	O1
19	O2

In the alpha (8–14 Hz) and beta (14–30 Hz) bands, we observed a spatial reorganization of connectivity rather than a uniform reduction. At retest, new network hubs emerged predominantly over right-lateralized and temporal regions—including T4, C4, T5, and F7—suggesting a shift in functional integration away from canonical midline hubs. This was particularly evident in the beta band, where we also observed a marked decoupling of the central Cz electrode, indicated by a clear reduction in its connectivity with the rest of the network. The emergence of similar hub locations in both alpha and beta bands suggests a stable reconfiguration of functional hubs, possibly reflecting compensatory recruitment of lateral sensorimotor or associative areas following TMS-induced disruption at Cz.

A particularly striking feature is the pronounced reduction in connectivity centered around Cz in the alpha and beta bands, visible as a distinct blue cross pattern in the connectivity matrices at retest. This pattern likely reflects a local desynchronization effect at the TMS stimulation site, disrupting alpha- and beta-band communication between this sensorimotor hub and the wider network. These connectivity disruptions align well with the observed decreases in the Hurst exponent at Fz and Pz, suggesting that TMS-induced modulation propagates through fronto-central-parietal circuits in a frequency-specific manner.

### 3.5. Result 4: Impact of Neuromodulation on Directional Connectivity in Midline Regions

To further refine our analysis and investigate the network-level effects of stimulation, we focused on changes in directional connectivity specifically among midline electrodes.

**Figure 5:**
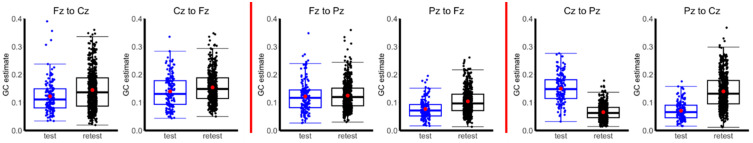
Directional connectivity (Granger causality estimates) between midline electrodes before and after rTMS: Boxplots below illustrate directional Granger causality (GC) estimates between midline electrode pairs during the test (pre-TMS, blue) and retest (post-TMS, black) sessions. Each subplot represents a specific direction of information flow (e.g., Fz → Cz, Cz → Pz). Individual data points are overlaid, and red circles indicate group means.

In terms of directional connectivity, no substantial changes were observed between Fz and Cz, nor between Fz and Pz, in either direction. In contrast, as Figure 5 mentioned, connectivity between Cz and Pz was significantly modulated following TMS. Specifically, there was a notable increase in bottom-up information flow from Pz to Cz and a decrease in top-down connectivity from Cz to Pz. These results suggest a directionally specific reorganization of midline connectivity, potentially reflecting enhanced posterior-to-central integration following low-frequency TMS at Cz. Such directional shifts align well with the previously observed changes in beta-band hubness, supporting the interpretation that TMS reduces central cortical dominance while enhancing posterior-driven network dynamics.

## 4. Discussion

This exploratory single-case study investigated how a mixed-frequency, EEG-guided rTMS protocol might influence multiscale cortical dynamics in a pre-adolescent with autism spectrum disorder (ASD). By combining spectral decomposition, long-range temporal correlation (LRTC) analysis, and connectivity metrics, we identified reproducible and frequency-specific alterations in cortical activity following stimulation. The principal findings included (i) increased delta and reduced alpha/beta power, (ii) steepening of the aperiodic slope and elevation of the offset—both consistent with increased inhibitory tone, (iii) reduced Hurst exponents at lower frequencies, (iv) redistribution of network hubs away from midline regions, and (v) reversal of directional connectivity between posterior and central midline areas. Collectively, these results illustrate that multiscale EEG biomarkers can sensitively capture cortical state changes induced by rTMS in ASD, without implying clinical efficacy or generalizability. Consistent with prior studies applying low-frequency or mixed-frequency rTMS, broad spectral changes were most evident over midline and central scalp regions [7,19,43,44].All procedures were conducted under institutional ethics oversight and with in-kind technical support from Cosmos Healthcare, which provided access to equipment and space but did not influence design, analysis, or interpretation.

### 4.1. Spectral Power and Topographical Reorganization: The Role of Spectral Parameterization in Clarifying Neuromodulatory Effects

Consistent with prior studies applying low-frequency or mixed-frequency rTMS, broad spectral changes were most evident over midline and central scalp regions [4,22,45], However, decomposition of the spectra into periodic (oscillatory) and aperiodic (1/f) components revealed that several apparent changes—particularly within the alpha and theta bands—were partly driven by alterations in the broadband background. Parameterization enabled isolation of genuine oscillatory modulations from broadband fluctuations, exposing patterns that would otherwise remain obscured. This methodological distinction underscores the necessity of separating periodic and aperiodic components when interpreting frequency-specific neuromodulatory effects [5].

Post-rTMS increases in both aperiodic exponent and offset, most prominently at Cz, suggest a shift toward lower-frequency-dominated activity and potentially greater inhibitory tone. Steeper slopes in EEG spectra have been linked to higher GABAergic inhibition or reduced cortical excitability [5,8]. Conversely, flatter slopes and elevated offsets have been reported in ASD and ADHD cohorts, often associated with cortical hyperexcitability and sensory hypersensitivity [10]. The observed steepening of the slope and normalization of offset therefore may represent a transient modulation of the underlying excitation–inhibition (E/I) balance, although causality cannot be inferred in a single-case design.

Importantly, periodic alpha power—which appeared reduced in the raw spectra—increased markedly after aperiodic adjustment, especially at Cz. This dissociation illustrates how changes in the broadband slope can mask true rhythmic effects. Enhanced alpha activity over central regions is frequently interpreted as a correlate of cortical inhibition or functional disengagement of sensorimotor areas [45,46]. In this context, the post-rTMS alpha enhancement may indicate a redistribution of spectral energy, with concurrent beta reductions suggesting a more balanced oscillatory regime.

A similar spatially differentiated pattern emerged in the theta band, with periodic theta increasing at Fz but decreasing at Cz and Pz. The frontal theta increase paralleled a rise in the theta/beta ratio (TBR) at Fz. Although elevated TBR is sometimes interpreted as an index of cortical underarousal or attentional inefficiency [47], its meaning depends strongly on cortical region, baseline state, and the direction of constituent band changes. Here, the TBR increase appears to arise from a combination of elevated theta and decreased beta, consistent with a relative normalization of frontal oscillatory dynamics rather than dysfunction. These patterns may reflect short-term adjustments in network gain or top-down control processes following rTMS-induced perturbation.

### 4.2. Long-Range Temporal Correlations (LRTC)

Detrended fluctuation analysis (DFA) revealed a general reduction in Hurst exponents within the 1–10 Hz range, with additional decreases in the 10–30 Hz band at Fz and Pz. Lower Hurst values indicate more stochastic, less temporally correlated activity interpreted in some models as a shift away from persistent excitation toward more dynamically regulated states [12,13]. In ASD, elevated long-range correlations have been associated with hyper-recurrent network loops, behavioral rigidity, and sensory hypersensitivity. The present reduction in Hurst exponents therefore may reflect a normalization of excessive temporal persistence, consistent with a move toward more adaptable network dynamics, although causality cannot be inferred from a single case.

Because LRTCs arise from the balance between recurrent excitation and inhibition, reduced temporal autocorrelations following rTMS could signify rebalancing of circuit-level persistence, especially in slower oscillatory bands that mediate large-scale coordination [48,49]. Regionally, the effect was more pronounced at Fz and Pz than at Cz, suggesting that associative frontoparietal cortices may be more responsive to low-frequency stimulation or possess greater adaptive plasticity. Alpha- and beta-band LRTC reductions at Fz and Pz further imply reorganization of temporal dynamics in circuits underlying executive control and sensory integration—processes often atypical in ASD. In contrast, no LRTC change above 30 Hz was detected at any site. Gamma activity, governed by local fast-timescale processing such as sensory encoding, typically shows weak temporal autocorrelation and limited sensitivity to slow neuromodulatory influences [50]. The absence of gamma-range effects is therefore consistent with the physiological bandwidth of rTMS and the central placement of stimulation sites.

### 4.3. Phase-Based Functional Connectivity

Phase-lag index (PLI) analysis revealed topological reorganization of functional networks, most prominently in the alpha and beta bands. Connectivity strength around the Cz node decreased, while lateral and associative regions showed relative increases, producing a lateral shift of network hubs. This pattern aligns with theoretical accounts of hub disruption in ASD, in which excessive central connectivity coexists with weakened long-range coordination [15,51]. The emergence of similar hub configurations across alpha and beta bands suggests a stable reconfiguration rather than a transient effect. The marked decoupling of Cz from surrounding nodes indicates reduced midline dominance and potentially greater engagement of distributed sensorimotor and associative networks. Such redistribution may support a more flexible, less rigid network topology, although further study is required to determine whether these shifts persist or generalize beyond this individual.

### 4.4. Connectivity and Information Flow

Granger-causality analysis demonstrated a directional reversal of information flow between midline electrodes following stimulation. Specifically, posterior-to-central (Pz → Cz) influence increased, while central-to-posterior (Cz → Pz) influence decreased. This shift parallels the reduction in frontal-beta hubness observed in PLI metrics and may indicate a temporary reduction of top-down dominance with enhanced posterior-driven integration. Although Cz and Pz provide a coarse approximation of hierarchical organization, this posterior-to-anterior redirection is consistent with developmental models in which efficient cognitive processing depends on balanced bottom-up and top-down communication—patterns often altered in ASD and ADHD [14,52,53]. No notable changes emerged in frontally mediated loops (Fz–Cz–Fz and Fz–Pz–Fz), suggesting spatial selectivity of the effect. This could reflect either relative resistance of frontal circuits to modulation or a ceiling effect in already hyperconnected regions. Overall, the directed-connectivity results complement the PLI findings, indicating that mixed-frequency rTMS can reorganize information-flow topology across midline networks within the limits of exploratory, single-subject inference. This study represents an exploratory single-subject, uncontrolled case, which substantially limits generalizability. Without a sham-TMS or cross-over condition, spontaneous neurodevelopmental changes, placebo effects, or contextual factors cannot be ruled out. Behavioral observations were restricted to parent reports and not corroborated by blinded or clinician-rated measures, introducing the possibility of expectancy or reporting bias.

All EEG data were obtained under eyes-open resting-state conditions; therefore, the findings may not extrapolate to task-evoked or cognitively engaged states. The four-month interval between baseline and follow-up assessments also introduces potential developmental or environmental confounds. The cumulative stimulation exposure (43,200 pulses) approaches the upper range of published pediatric safety recommendations, emphasizing the need for replication studies with lower or adaptively titrated dosing paradigms. Although no adverse events occurred, safety conclusions should remain conservative until validated in larger controlled samples. EEG data quality was rated low at baseline and very low at follow-up, largely due to electromyographic (EMG) contamination. Despite application of the MADE–FASTER automated preprocessing pipeline—which retained ≥6 min of artifact-free data per session—high-frequency estimates (beta and gamma bands) may remain partially biased. Future work should employ higher-density recordings, improved artifact control, and cross-modal validation (e.g., MEG or fNIRS) to strengthen reproducibility.

## 5. Conclusions

This proof-of-concept case study provides preliminary evidence that an EEG-guided, mixed-frequency rTMS protocol can induce measurable, multiscale modulation of cortical dynamics in a child with autism spectrum disorder. Across spectral, temporal, and connectivity domains, descriptive analyses revealed coordinated shifts in oscillatory power, aperiodic slope, long-range temporal correlations, and directional connectivity. While no causal or therapeutic inferences can be drawn, the convergence of these neurophysiological markers suggests that quantitative EEG (qEEG)-based monitoring can sensitively capture the immediate systems-level effects of rTMS. These findings underscore the feasibility—and methodological value—of integrating spectral parameterization, LRTC, and connectivity metrics into individualized neuromodulation research pipelines. Further controlled, multi-participant studies are needed to determine the reliability, safety, and clinical relevance of such EEG-guided stimulation approaches in ASD and other neurodevelopmental conditions.

## Figures and Tables

**Table 1:** EEG-guided rTMS stimulation parameters. Stimulation sites were selected based on baseline quantitative EEG (qEEG) abnormalities rather than standardized landmarks. Each row lists the 10–20 electrode sites, targeted network, stimulation frequency, total pulses, intensity relative to resting motor threshold (%RMT), and corresponding qEEG findings. Downward (↓) and upward (↑) arrows indicate reduced or increased values of the given metric, respectively.

Site (10–20)	Intended Network	Frequency	Total Pulses	%RMT	Baseline qEEG Finding
FP1 & FP2	Default-mode/vmPFC	10 Hz	600	100	Hypoconnectivity (↓beta)
F3	Left DLPFC	10 Hz	600	100	Reduced frontal beta power
F8	Right IFG	1 Hz	600	90	Elevated synchrony/connectivity
FZ	SMA/Midline	1 Hz	900	90	High beta & ↑aperiodic offset
CZ & PZ	Sensorimotor hubs	1 Hz	1200	90	↑Offset, ↓slope (hyperexcitability)
T6	pSTS	10 Hz	600	100	Low beta-2 (↓biological motion)

**Table 2:** Summary of adverse events observed during EEG-guided mixed-frequency rTMS (CTCAE v5.0). All adverse events were Grade 1 (mild), transient, and resolved without sequelae.

Adverse Event (Clinical Description)	CTCAE v5.0 Preferred Term	Grade	N Sessions (of 8)	Management	Outcome/Resolution Time	Relationship to rTMS †
Scalp discomfort at stimulation site	Pain, head	1	2	Short pause, reassurance; no medication	Resolved within minutes during session	Probably related
Transient mild headache after session	Headache	1	1	Rest at home; no specific treatment	Resolved within a few hours	Possibly related
Transient fatigue/drowsiness post-session	Fatigue	1	2	Rest, reduced activity on same day	Resolved by the following morning	Possibly related
Brief irritability/mood lability same day	Irritability	1	1	Supportive reassurance; no medication	Resolved by evening of the same day	Possibly related

† Relationship judged by the treating clinician (e.g., “possibly related”, “probably related”).

**Table 3:** Quantitative changes in the aperiodic components of the PSD before and after rTMS. Exponent—slope of the power spectrum, balance of E/I network. Offset—broadband shift of power across frequencies.

Metric	Channels	Test	Retest	% Increase
**Exponent (Slope)**	Fz	1.90	2.05	7.89
	Cz	1.82	2.52	38.48
	Pz	1.87	2.24	19.78
**Offset**	Fz	1.50	2.03	35.33
	Cz	1.35	2.58	91.11
	Pz	1.94	2.74	41.28

## Data Availability

Anonymized EEG data and analysis scripts are available from the corresponding author on reasonable request, subject to privacy safeguards and institutional approvals.
